# Radiation-Induced Liver Injury in Three-Dimensional Conformal Radiation Therapy (3D-CRT) for Postoperative or Locoregional Recurrent Gastric Cancer: Risk Factors and Dose Limitations

**DOI:** 10.1371/journal.pone.0136288

**Published:** 2015-08-20

**Authors:** Guichao Li, Jiazhou Wang, Weigang Hu, Zhen Zhang

**Affiliations:** Department of Radiation Oncology, Fudan University Shanghai Cancer Center and Department of Oncology, Shanghai Medical College, Fudan University, Shanghai, China; National Taiwan University, TAIWAN

## Abstract

**Purpose:**

This study examined the status of radiation-induced liver injury in adjuvant or palliative gastric cancer radiation therapy (RT), identified risk factors of radiation-induced liver injury in gastric cancer RT, analysed the dose-volume effects of liver injury, and developed a liver dose limitation reference for gastric cancer RT.

**Methods and Materials:**

Data for 56 post-operative gastric cancer patients and 6 locoregional recurrent gastric cancer patients treated with three-dimensional conformal radiation therapy (3D-CRT) or intensity-modulated radiation therapy (IMRT) from Sep 2007 to Sep 2009 were analysed. Forty patients (65%) were administered concurrent chemotherapy. Pre- and post-radiation chemotherapy were given to 61 patients and 43 patients, respectively. The radiation dose was 45–50.4 Gy in 25–28 fractions. Clinical parameters, including gender, age, hepatic B virus status, concurrent chemotherapy, and the total number of chemotherapy cycles, were included in the analysis. Univariate analyses with a non-parametric rank test (Mann–Whitney test) and logistic regression test and a multivariate analysis using a logistic regression test were completed. We also analysed the correlation between RT and the changes in serum chemistry parameters [including total bilirubin, (TB), direct bilirubin (D-TB), alkaline phosphatase (ALP), alanine aminotransferase (ALT), aspartate aminotransferase (AST) and serum albumin (ALB)] after RT.

**Results:**

The Child-Pugh grade progressed from grade A to grade B after radiotherapy in 10 patients. A total of 16 cases of classic radiation-induced liver disease (RILD) were observed, and 2 patients had both Child-Pugh grade progression and classic RILD. No cases of non-classic radiation liver injury occurred in the study population. Among the tested clinical parameters, the total number of chemotherapy cycles correlated with liver function injury. V35 and ALP levels were significant predictive factors for radiation liver injury.

**Conclusions:**

In 3D-CRT for gastric cancer patients, radiation-induced liver injury may occur and affect the overall treatment plan. The total number of chemotherapy cycles correlated with liver function injury, and V35 and ALP are significant predictive factors for radiation-induced liver injury. Our dose limitation reference for liver protection is feasible.

## Introduction

Gastric cancer is one of the most common cancers in China, and patients with lymph node-positive disease have a 5-year survival rate as low as 15–20%. Even node-negative patients have a 5-year survival rate of only 45–55% if the T stage is advanced (T3-T4N0) [1]. Three-dimensional radiation therapy (3D-RT) is a common treatment method for gastric adenocarcinoma [2]. However, the radiation volume for gastric cancer is very large, including the clinical target volume (CTV) that is adjacent to the liver and contains the tumour bed, anastomosis, gastric remnant (pT3-T4), and regional draining lymph nodes. Furthermore, a larger margin should be added to the CTV for the respiratory motion and inter-fractional variability, thus increasing the planning tumour volume (PTV). Additionally, the liver occupies a large proportion of the upper abdominal cavity, and hepatic hilar lymph nodes need to be contained in the CTV for the majority of patients. The liver inevitably receives a high dose in post-operative or locoregional recurrence gastric cancer patients. Thus, it is difficult to deliver an effective prescription dose to the target while keeping the exposure of normal tissue (e.g., liver and kidney) to a low dose.

Moreover, patients with advanced stages of cancer require radiation therapy (RT) and chemotherapy. The advantage of oral chemotherapy drugs, such as capecitabine and S-1, has been shown in a randomized clinical trial [3–5]; however, the safety profile of both oral drugs includes liver toxicity. Thus, the combination of these chemotherapy drugs and radiotherapy can induce severe liver injury [6]. Radiation-induced liver injury may affect the treatment plan. Thus, it is important to protect the liver from radiation injury during RT. The normal tissue tolerance of the liver reported in the NCCN guideline is V30<60% for gastric cancer radiotherapy; however, this large value may result in liver injury. A more detailed dose-limitation reference for the RT for gastric cancer patients is urgently needed.

A number of models have been used to predict the risk of radiation-related liver injury. The probability of radiation-induced liver disease (RILD) using the Lyman-Kutcher-Burman model of normal tissue complication probability (NTCP) for primary liver cancer (PLC) treated with three-dimensional conformal radiation therapy (3D-CRT) was established by Dawson et al. and Xu et al. [7,8]. The relationship between the mean liver dose and RILD was also provided. Other models, such as dose-volume effect [9,10], artificial neural networks [11] and parallel-type organ damage [12,13], have also been used to estimate RILD. These previous studies assessed liver cancer, and the findings may not apply to patients with gastric cancer. Unfortunately, there are fewer reports on the relationship between dose distribution and radiation-related liver complications in patients with gastric cancer.

The aims of current study were to investigate the predictive factors of radiation-induced liver injury in adjuvant and palliative gastric cancer RT, analyse the dose-volume effects of liver injury in gastric cancer RT, and offer a dose limitation reference for gastric cancer RT.

## Methods and Materials

### Patients and Treatment

All data were collected from consenting individuals according to the protocols approved by the Ethics Review Board at the Fudan University Shanghai Cancer Center. The consents were verbal, and we recorded the participant consents on our follow-up record to confirm that he/she wanted to join the study. The ethics committee approved this consent procedure. From Sep 2007 to Sep 2009, a total of 90 consecutive and non-selected gastric cancer patients underwent post-operative adjuvant or palliative RT at the Fudan University Shanghai Cancer Center. Among them, 76 patients with locally advanced post-operative gastric cancer and 14 patients with locoregional recurrence and non-metastatic gastric cancer patients underwent radiotherapy. Twenty-eight patients were excluded from the current study due to incomplete dosimetric parameters (12 patients) or insufficient follow-up data (16 patients). Thus, the data from a total of 62 patients were included in this study, including 56 post-operative patients who underwent adjuvant chemoradiotherapy and 6 locoregional recurrent patients who received palliative chemoradiotherapy. Out of this total, 45 patients were male, and 17 patients were female, with a median age of 56 years (range, 32–78 years). Forty patients (65%) were administered concurrent chemotherapy, including 21 patients given capecitabine and 19 patients given 5-FU. Pre- and post-radiation chemotherapy were administered to 61 patients and 43 patients, respectively, with 1–10 (median 5) cycles and a chemotherapy regimen of ECF or modified ECF (EOF or EOX).

### Radiation Therapy

For post-operative patients, the target volume for RT included the tumour bed, anastomosis, gastric remnant (pT3-T4), and regional draining lymph nodes (e.g., para-aortic and paracaval lymph nodes, celiac axis, splenic artery and hepatic artery). The target volume was determined from the pre-operative CT scan, surgical clips, and barium examination. For patients with locoregional recurrence, the target volume for RT included the recurrent lesions only and was determined from the enhanced-contrast CT scan. Treatment was delivered using 3D-CRT or intensity-modulated radiation therapy (IMRT) technology. Respiratory movement was estimated by fluoroscopy and repeated CT scanning. The PTV was an expansion of CTV because of the respiratory motion and inter-fractional uncertainty. Active Breathing Coordinator (ABC) technology was used to reduce organ movement in patients with a large displacement (>1 cm) due to breathing. The prescription dose was 45–50.4 Gy in 25–28 fractions. Three-dimensional dose calculations were performed using Pinnacle 3 (ADAC, Milpitas, CA, USA) with a tissue density inhomogeneity correction. The goal of the treatment plan was to ensure that the prescription dose covered at least 95% of the PTV and that 95% of the prescription dose covered at least 99% of the PTV. The dose constraints for the organs at risk (OAR) included each kidney V15 (volume receiving ≥15 Gy) <50%, a mean dose of each kidney <16 Gy, liver V30 (volume receiving ≥30 Gy) <30%, a mean liver dose (MLD) less than 23 Gy and a spinal cord maximum dose <45 Gy. If the aim of the treatment plan could not be met because of geometry size, position of the PTV or critical structures, a compromise was made for clinical purposes. Radiation therapy was delivered with a 6-MV linear accelerator, and IMRT was delivered with a step-and-shoot technique.

### Evaluation of Liver Function and Follow-Up

All patients were required to undergo follow-up according to the protocol, including weekly meetings during RT and at least 4 monthly meetings after treatment completion. Follow-up examinations, including history and physical examination, were routinely performed, and all patients were evaluated for liver function, including bilirubin (total and direct, TB, D-TB), alanine aminotransferase (ALT), aspartate aminotransferase (AST), alkaline phosphatase (ALP) and serum albumin (ALB). Baseline liver functions and post-RT changes in liver function were evaluated with the Child-Pugh score system. We recorded the classic and non-classic RILD cases according to the definition of Pan et al. [14]. We defined liver function injury as a progression in the patient’s Child-Pugh score.

### Statistical Analyses

#### Data analysis

Clinical parameters, including gender, age, hepatic B virus status, concurrent chemotherapy, and the total number of chemotherapy cycles, were used in the analysis, and the dose volume histogram (DVH) of the treatment plan was recorded for each patient. The fractions of the liver receiving more than 5, 10, 15, 20, 30, 35, 40, and 45 Gy, MLD and maximal dose to the liver were computed for each patient from the liver DVH.

A univariate analysis using a non-parametric rank test (Mann–Whitney test) and logistic regression test and a multivariate analysis using the logistic regression test were completed. A p-value of 0.05 was considered significant for the rank tests and logistic analysis. We analysed the correlation between RT and the changes in serum chemistry parameters (including TB, D-TB, ALP, ALT, AST and ALB) after RT.

## Results

Out of the 62 patients, 58 patients were Child-Pugh grade A, and 4 patients were Child-Pugh grade B before RT. Fourteen patients were Child-Pugh grade B, and 45 patients were Child-Pugh grade A after RT. The Child-Pugh grade progressed from grade A to grade B after RT in 10 patients. A total of 16 cases of classic RILD (with elevation of ALP only) were observed. Two patients had both Child-Pugh grade progression and classic RILD (with elevation of ALP only). Non-classic radiation liver injury was not observed in any of the patients.

The results of the univariate analysis evaluating the association between clinical and dose-volumetric parameters are summarized in Table 1 and Table 2. Among the studied clinical parameters, the total number of chemotherapy cycles correlated with liver dysfunction. Among the dose-volumetric parameters, V30-V40 was associated with liver function injury, and V35 was the most predictive factor. The Spearman’s rank correlation coefficients between DVH parameters are shown in Table 3. Close correlations among DVH parameters were found, especially in V30-V40, which was significant in the univariate analysis. Thus, we chose V35 as the dosimeter factor to complete a multivariate analysis.

**Table 1:** Univariate analysis of clinical factors related to liver function injury.

Characteristics		Without RILD	With RILD	P-value
**Gender**	Male	25	20	0.854
	Female	9	8	
**Age**	≥60	7	12	0.063
	<60	27	16	
**Concurrent chemotherapy**	With	21	19	0.618
	Without	13	9	
**Hepatitis B virus**	Positive	4	0	1.000
	Negative	30	28	
**Chemotherapy cycle number**		4.3±2.3	5.3±1.8	0.036

*A non-parametric rank test (Mann–Whitney U test) was used for continuous variables, and a logistics regression analysis was used for binary parameters.

**Table 2:** Univariate analysis of dose-volumetric factors related to liver function injury.

Parameter	Without RILD	With RILD	P-value
V5 (%)	85.5±23.4	93.2±11.6	0.783
V10 (%)	60.9±23.2	67.1±14.9	0.666
V15 (%)	44.1±17.8	48.9±12.5	0.676
V20 (%)	33.4±15.6	39.3±10.4	0.181
V25 (%)	25.8±12.9	31.9±8.8	0.112
V30 (%)	19.8±10.7	25.7±7.3	0.042
V35 (%)	15.8±9	20.7±6.1	0.030
V40 (%)	12.5±7.7	16.2±5.3	0.038
V45 (%)	7.1±5	8.0±4.2	0.319
Mean dose (cGy)	1760±589	1991±340	0.159
Max dose (cGy)	4583±1094	4950±164	0.475

Abbreviations: Vx represents the percentage of liver volume receiving more than x Gy. The data are presented as the number of patients or mean±standard deviation.

*A non-parametric rank test (Mann–Whitney U test) was used for continuous variables.

**Table 3:** Spearman’s rank correlation between DVH parameters.

	V5	V10	V15	V20	V25	V30	V35	V40	V45	Mean dose	Max dose
**V5**	-										
**V10**	0.762	-									
**V15**	0.685	0.821	-								
**V20**	0.559	0.631	0.908	-							
**V25**	0.498	0.528	0.763	0.918	-						
**V30**	0.386	0.374	0.579	0.784	0.921	-					
**V35**	0.313	0.295	0.483	0.702	0.839	0.972	-				
**V40**	0.260	0.273	0.436	0.662	0.799	0.930	0.973	-			
**V45**	0.244*	0.191*	0.239	0.405	0.523	0.663	0.730	0.811	-		
**Mean dose**	0.643	0.686	0.855	0.929	0.937	0.868	0.804	0.767	0.532	-	
**Max dose**	0.219*	0.244*	0.305	0.315	0.296	0.331	0.364	0.397	0.599	0.364	-

The abbreviations are the same as in Table 2.

*The correlation is not significant at the 0.05 level (2-tailed).

**Fig 1:**
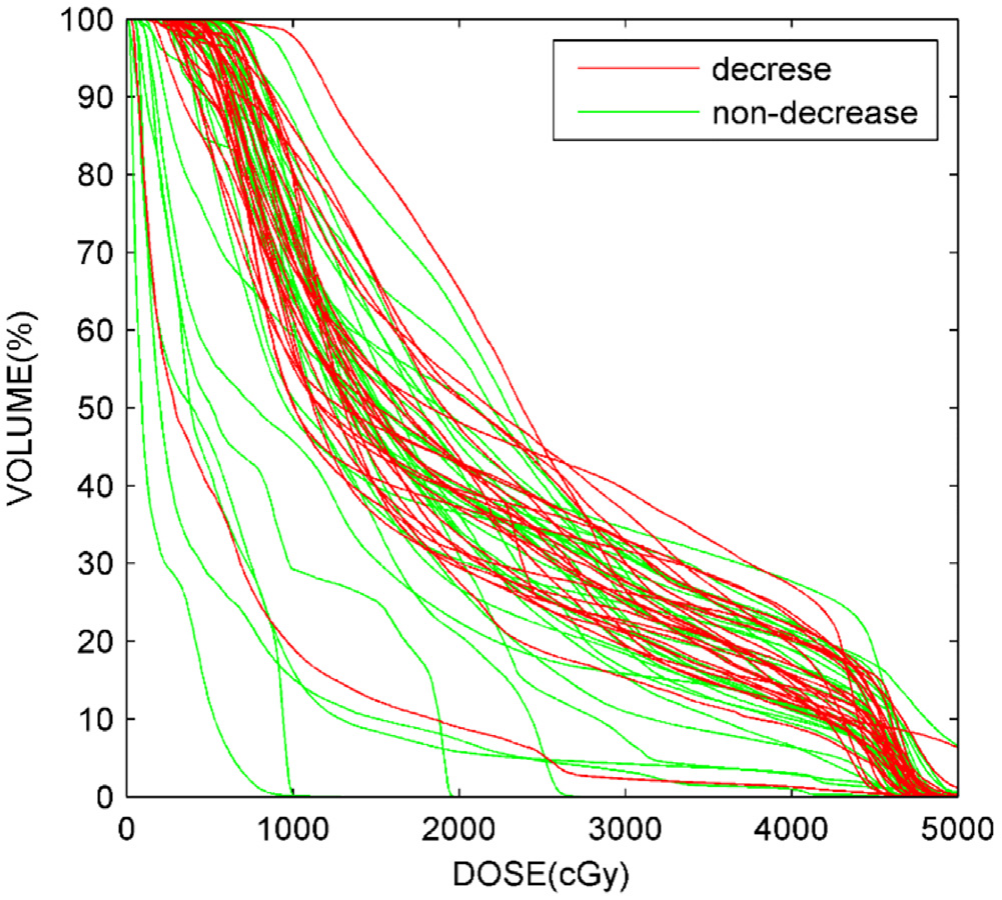
Characteristic normal liver DVHs for all patients with or without radiation-induced liver injury.

**Fig 2:**
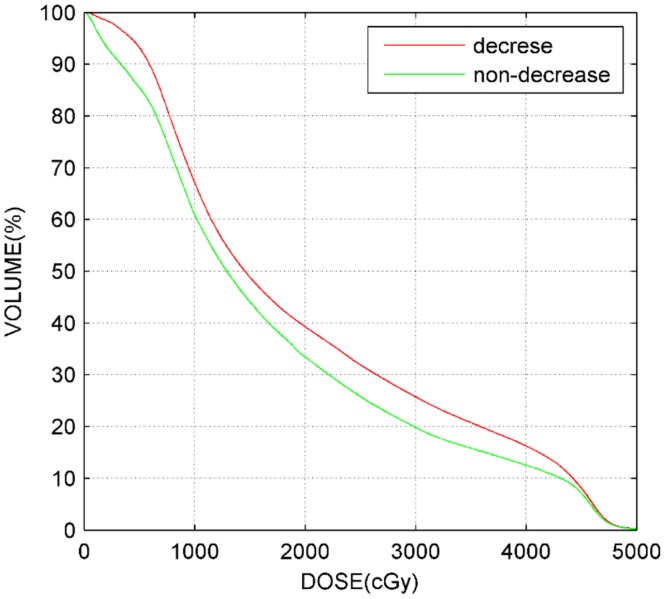
Characteristic normal liver mean DVHs for patients with or without radiation-induced liver injury.

A multivariate analysis (Table 4) was performed for liver function injury using the 3 variables with the most significant p-values in the univariate analysis. In the multivariate analysis, V35 was the only significant parameter associated with liver function injury. The DVHs of all 62 patients and mean DVHs of patients with or without liver injury are plotted in Fig 1 and Fig 2. The shapes of the DVH curves varied greatly among patients.

**Table 4:** Multivariate analysis.

Parameter	P-value*
**V35**	0.03
**Age**	0.94
**Chemotherapy cycle number**	0.14

The abbreviations are the same as in Table 2.

*multivariate logistic regression analysis

We analysed the correlation between dose-volumetric parameters and abnormal serum chemistry parameters (TB, D-TB, ALP, ALT, AST and ALB) after RT. We found that AST, D-TB and TB were not related to delivery dose. The relationship between the other three parameters and the DVH parameters is summarized in Table 5. The outcome for ALB was similar to the analysis based on the Child-Pugh score system. V35 and ALP were significant predictive factors for radiation-induced liver injury.

**Table 5:** The relationship between three parameters (ALB, ALT and ALP) and DVHs.

Parameters	P-value		
	ALB	ALT	ALP
**V3.5**	0.884	0.030	0.008
**V5**	0.763	0.339	0.105
**V10**	0.379	0.848	0.575
**V15**	0.414	0.988	0.328
**V20**	0.250	0.627	0.158
**V25**	0.396	0.566	0.057
**V30**	0.088	0.941	0.073
**V35**	0.050	0.670	0.089
**V40**	0.056	0.670	0.270
**V45**	0.056	0.627	0.758
**Mean dose**	0.225	0.780	0.115
**Max dose**	0.281	0.206	0.250

The abbreviations are the same as in Table 2.

*non-parametric rank test (Mann–Whitney U test)

## Discussion

This is the first investigation reporting the correlation between clinical parameters, DVH and liver function injury in gastric cancer RT. Our study showed that radiation-induced liver injury occurred in 3D-CRT for gastric cancer patients. Among the assessed clinical parameters, the total number of chemotherapy cycles correlated with liver function injury, and V35 and ALP were significant predictive factors for radiation-induced liver injury.

The majority of previously applied normal liver tissue constraints in radiotherapy planning were based on the studies of liver toxicity after radiotherapy for primary or metastatic liver cancer. The radiation-induced hepatic injury differs between liver cancer and gastric cancer patients. More patients with gastric cancer had normal liver function before radiotherapy, and the dose distribution in the liver varied. Moreover, the treatment strategies and methods differ between liver cancer and gastric cancer patients. This straightforward study aimed to correlate clinical outcome with irradiated liver volume and increase the details for the liver tolerance guidelines. Thus, this study has merit.

Our results showed that the response to liver radiation in gastric cancer RT was not as strong as in liver cancer RT. For whole liver RT, the prescription dose was limited to the whole-liver tolerance dose, which corresponded with a 5% risk of RILD after a whole-liver dose delivery of 30–35 Gy in 2 Gy per fraction [15]. For partial liver RT, several studies noted a dosimetric parameter associated with increased toxicity risk, including the mean liver dose and V30 [9,16–19]. In our study, several DVH parameters, such as V30, V35, and V40, were predictors of liver function injury. However, these DVH parameters correlated with each other, and it was difficult to select the most valuable parameter for predicting the incidence of liver function injury. We chose V35 because it was the most significant predictive parameter. The mean liver dose was not significantly associated with liver function deterioration. This finding may be due to the varied DVH profile among patients.

The evaluation criterion used was different for patients with liver cancer and gastric cancer. Liver cancer, the most common liver toxicity after RT, includes a clinical syndrome of anicteric hepatomegaly, ascites, and elevated liver enzymes (ALP more than the transaminases) occurring typically between 2 weeks to 3 months after completion of RT. Most research uses the Cancer Therapy Evaluation Program and Common Terminology Criteria for Adverse Events (CTCAE) to evaluate liver toxicity after RT. The liver function of the majority of patients with gastric cancer was better compared with patients with liver cancer. The high-dose delivery to the liver in gastric cancer RT was less, and none of the patients suffered from serious liver toxicity in the current study.

The dose distribution in liver during gastric cancer RT differs from liver cancer RT. Sparing the normal liver in patients with liver cancer was more difficult. In gastric RT, IMRT technology can be used to spare more liver volume, which may be beneficial for patients. In liver radiation, the target of radiation is always surrounded by normal liver parenchyma, which inevitably increases the normal liver dose. According to Jackson’s report, the liver was composed of independent functional subunits; thus, the distribution of dose may influence liver function [13].

The relationship between treatment planning and serum chemistry parameters was established by recording and analysing dosimetric and clinical data. Our results show 2 effects of radiation. First, ALT and ALP were sensitive to a low-dose radiation volume if the volume of 3.5 Gy covered nearly the whole liver; therefore, these two parameters will reach abnormal levels after radiation. This result indicates that ensuring a part of liver does not receive radiation during the therapy may decrease the incidence of liver toxicity. This phenomenon was also described by Liang et al. [17]. We also found that ALB results were similar to the Child-Pugh scores. The varying results for ALB and ALP may represent different mechanisms or models of liver injury that correspond to acute and chronic liver injury from early- and late-responding liver tissue.

Many studies have used NTCP to estimate the risk of radiation-related liver complications. This method assumes a sigmoidal relationship between the dose of uniform radiation given to a volume of an organ and the chance of a complication occurring [20,21]. NTCP provides a quantitative biophysical measure of a dose distribution; however, the predictive power of NTCP models has not yet been proven clinically. Compared with other liver radiation therapy studies, we used stricter dose limitations for the liver; however, we still observed radiation-induced liver injury. This finding may be because most of the patients received several cycles of chemotherapy and concurrent chemotherapy, which can render patients more susceptible to RT-induced liver injury. Therefore, despite well-functioning pre-treatment livers in gastric cancer patients, we should protect the liver from radiation-induced liver injury to prevent the delay of chemotherapy. In our study, all of the patients who developed liver dysfunction recovered after treatment, indicating that our dose limitations for the liver is safe and should be recommended for gastric cancer patients.

We consider our results far from conclusive, and several important issues need to be addressed. First, the determination of the dose limitations for radiation therapy for gastric cancer patients is limited because of the retrospective study design. Second, the patient characteristics are not homogeneous in this study. Third, from a modelling point of view, this is a unique situation because of the wide spectrum of DVH shapes; however, this variability requires more patients to be enrolled in the study. Lastly, the chemotherapy regimen and cycles differed among patients, and the observation of a significant relationship between the number of chemotherapy cycles and liver dysfunction may have been influenced by confounding non-radiation-based factors. More clinical trials, preferably in a prospective design, are needed to determine the threshold value of the DVH parameters that can predict liver function.

## Supporting Information

S1 Table (DOC)
