# Supplementary material for: Radiation-Induced Liver Injury in Three-Dimensional Conformal Radiation Therapy (3D-CRT) for Postoperative or Locoregional Recurrent Gastric Cancer: Risk Factors and Dose Limitations
Source: PLoS One. 2015 Aug 20;10(8):e0136288. doi: 10.1371/journal.pone.0136288 (PMC4546190; doi:10.1371/journal.pone.0136288)
Supplement: S1 Table — (DOC) [file pone.0136288.s001.doc]

Table 1.Univriate analysis of clinical factors related to liver function injury

| Characterize | Without RILD | With RILD | P-value |
| --- | --- | --- | --- |
| Gender |  |  | 0.854 |
| Male | 25 | 20 |  |
| Female | 9 | 8 |  |
| Age |  |  | 0.063 |
| >=60 | 7 | 12 |  |
| <60 | 27 | 16 |  |
| Concurrent chemotherapy |  |  | 0.618 |
| With | 21 | 19 |  |
| Without | 13 | 9 |  |
| Hepatitis B virus |  |  | 1.000 |
| Positive | 4 | 0 |  |
| Negative | 30 | 28 |  |
| chemotherapy cycle number | 4.3±2.3 | 5.3±1.8 | 0.036 |

*non-parametric rank test (Mann–Whitney U test) for continuously variable parameters and logistics regression analysis for binary parameters.

Table 2.Univriate analysis of dose-volumetric factors related to liver function injury

| Characterize | Without RILD | With RILD | p-value |
| --- | --- | --- | --- |
| V5(%) | 85.5±23.4 | 93.2±11.6 | 0.783 |
| V10(%) | 60.9±23.2 | 67.1±14.9 | 0.666 |
| V15(%) | 44.1±17.8 | 48.9±12.5 | 0.676 |
| V20(%) | 33.4±15.6 | 39.3±10.4 | 0.181 |
| V25(%) | 25.8±12.9 | 31.9±8.8 | 0.112 |
| V30(%) | 19.8±10.7 | 25.7±7.3 | 0.042 |
| V35(%) | 15.8±9 | 20.7±6.1 | 0.030 |
| V40(%) | 12.5±7.7 | 16.2±5.3 | 0.038 |
| V45(%) | 7.1±5 | 8.0±4.2 | 0.319 |
| mean dose(cGy) | 1760±589 | 1991±340 | 0.159 |
| max dose(cGy) | 4583±1094 | 4950±164 | 0.475 |

Abbreviations: Vx represents the percentage of liver volume receiving more than x Gy.

Data presented as number of patients or mean ± standard deviation.

*non-parametric rank test (Mann–Whitney U test) for continuously variable parameters.

Table 3. Spearman’s rank correlation between DVH parameters

|  | V5 | V10 | V15 | V20 | V25 | V30 | V35 | V40 | V45 | mean dose | max dose |
| --- | --- | --- | --- | --- | --- | --- | --- | --- | --- | --- | --- |
| V5 | - |  |  |  |  |  |  |  |  |  |  |
| V10 | 0.762 | - |  |  |  |  |  |  |  |  |  |
| V15 | 0.685 | 0.821 | - |  |  |  |  |  |  |  |  |
| V20 | 0.559 | 0.631 | 0.908 | - |  |  |  |  |  |  |  |
| V25 | 0.498 | 0.528 | 0.763 | 0.918 | - |  |  |  |  |  |  |
| V30 | 0.386 | 0.374 | 0.579 | 0.784 | 0.921 | - |  |  |  |  |  |
| V35 | 0.313 | 0.295 | 0.483 | 0.702 | 0.839 | 0.972 | - |  |  |  |  |
| V40 | 0.260 | 0.273 | 0.436 | 0.662 | 0.799 | 0.930 | 0.973 | - |  |  |  |
| V45 | 0.244* | 0.191* | 0.239 | 0.405 | 0.523 | 0.663 | 0.730 | 0.811 | - |  |  |
| mean dose | 0.643 | 0.686 | 0.855 | 0.929 | 0.937 | 0.868 | 0.804 | 0.767 | 0.532 | - |  |
| max dose | 0.219* | 0.244* | 0.305 | 0.315 | 0.296 | 0.331 | 0.364 | 0.397 | 0.599 | 0.364 | - |

Abbreviations as in Table 2.

*Correlation isn’t significant at the 0.05 level (2-tailed).

Table 4. Multivariate analysis

| Characterize | p-value* |
| --- | --- |
| V35 | 0.03 |
| Age | 0.94 |
| chemotherapy cycle number | 0.14 |

Abbreviations as in Table 2.

*multivariate logistic regression analysis

Table 5. The relationship between the three parameters (ALB, ALT and ALP) and DVH parameters

| Parameters | p-value | | |
| --- | --- | --- | --- |
|  | ALB | ALT | ALP |
| V3.5 | 0.884 | 0.030 | 0.008 |
| V5 | 0.763 | 0.339 | 0.105 |
| V10 | 0.379 | 0.848 | 0.575 |
| V15 | 0.414 | 0.988 | 0.328 |
| V20 | 0.250 | 0.627 | 0.158 |
| V25 | 0.396 | 0.566 | 0.057 |
| V30 | 0.088 | 0.941 | 0.073 |
| V35 | 0.050 | 0.670 | 0.089 |
| V40 | 0.056 | 0.670 | 0.270 |
| V45 | 0.056 | 0.627 | 0.758 |
| mean dose | 0.225 | 0.780 | 0.115 |
| max dose | 0.281 | 0.206 | 0.250 |

Abbreviations as in Table 2.

*non-parametric rank test (Mann–Whitney U test)
